# Support From a Distance: How Home Care Agencies Influence Paid Caregiving in the Home

**DOI:** 10.1093/geroni/igab046.833

**Published:** 2021-12-17

**Authors:** Emily Franzosa, Robyn Stone

## Abstract

Paid caregivers (e.g., home health aides, personal care attendants, and other direct care workers) who care for functionally impaired older adults in the home frequently report that while rewarding, their work is logistically, physically, and emotionally demanding. Unlike direct care workers in institutional settings, paid caregivers work with care recipients one-on-one in private settings and often have limited contact with or support from their employers. These factors contribute to high workforce turnover and may impact the quality of patient care. In this symposium, we explore ways that home care agency policies and practices influence the experience of giving and receiving care in the home. First, Bryant et al. describe the range of agency-based models and the impact of workplace design in creating supportive working environments. Next, Fabius et al. explore characteristics of direct care agencies across Maryland, with implications for worker training and support. Reckrey et al. describe the differing perceptions of aides, caregivers and providers around the role agencies play in defining paid caregivers’ roles, and how this may lead to conflict within the caregiving team. Finally, in the context of COVID-19, Franzosa et al. examine communication and coordination between Veterans Affairs-paid agencies and home health aides during the pandemic, while Tsui et al. present a case study of an agency’s efforts to support paid caregivers through group support calls. Together, these studies highlight challenges in the structure, organization and perceptions of home care agencies, and identify potential avenues for agencies to support paid caregivers and their clients.

